# Precise and efficient genome editing in zebrafish using the CRISPR/Cas9 system

**DOI:** 10.1242/dev.115584

**Published:** 2014-12-15

**Authors:** Uwe Irion, Jana Krauss, Christiane Nüsslein-Volhard

**Affiliations:** Max-Planck-Institut für Entwicklungsbiologie, Spemannstr. 35, Tübingen 72076, Germany

**Keywords:** Zebrafish, Genome editing, *albino*, *slc45a2*, CRISPR/Cas

## Abstract

The introduction of engineered site-specific DNA endonucleases has brought precise genome editing in many model organisms and human cells into the realm of possibility. In zebrafish, loss-of-function alleles have been successfully produced; however, germ line transmission of functional targeted knock-ins of protein tags or of SNP exchanges have not been reported. Here we show by phenotypic rescue that the CRISPR/Cas system can be used to target and repair a premature stop codon at the *albino* (*alb*) locus in zebrafish with high efficiency and precision. Using circular donor DNA containing CRISPR target sites we obtain close to 50% of larvae with precise homology-directed repair of the *alb^b4^* mutation, a small fraction of which transmitted the repaired allele in the germ line to the next generation (3/28 adult fish). The *in vivo* demonstration of germ line transmission of a precise SNP exchange in zebrafish underscores its suitability as a model for genetic research.

## INTRODUCTION

Zinc-finger nucleases (ZNFs) (Durai et al., 2005), transcription activator-like effector nucleases (TALENs) (Cermak et al., 2011) and a system based on the prokaryotic clustered, regularly interspaced short palindromic repeats (CRISPR) and the CRISPR-associated proteins (Cas) (Jinek et al., 2012) have recently been established as efficient tools for gene disruptions in many species (reviewed by Gaj et al., 2013). All three methods are based on the targeted introduction of DNA double-strand breaks into the genome. Repair of these breaks by non-homologous end joining is error prone and frequently leads to small insertions or deletions (indels), giving rise to loss-of-function mutations by frameshifts.

In zebrafish, the CRISPR/Cas system can generate gene knockouts with very high frequency (75-99%) (Cade et al., 2012; Hwang et al., 2013). Targeted knock-ins, however, are still difficult and the reported efficiencies, based on PCR analyses or next generation sequencing, are fairly low (Auer and Del Bene, 2014). Owing to imprecise targeting and the introduction of additional mutations, the resulting fish frequently do not display the anticipated phenotypic characteristics, nor has the propagation of a functional knock-in in the germ line of zebrafish been documented (Zu et al., 2013; Auer et al., 2014; Hruscha et al., 2013). This is in contrast to cultured human cells and mouse embryonic stem cells or zygotes, where the CRISPR/Cas system has been used together with single-stranded oligodeoxynucleotides (ssODNs) or conventional targeting vectors as donors for homology-directed repair (HDR), thereby allowing precise single nucleotide polymorphism (SNP) exchange, the knock-in of small affinity tags (HA, FLAG), of loxP sites, and of larger fluorescent protein tags (Gonzalez et al., 2014; Wang et al., 2013; Yang et al., 2013).

Here we used the *albino* (*alb*; *slc45a2*) locus in zebrafish to assess the potential of the CRISPR/Cas system to induce SNP exchanges by HDR after co-injection with appropriate donor DNAs. *alb* mutant larvae are pale, as the melanophores are unable to produce melanin. We show repair of the *alb^b4^* mutation, as demonstrated by the appearance of pigmented cells in the larvae, when wild-type donor DNA is co-injected with the CRISPR/Cas system at the one-cell stage. The frequency of repair is enhanced by several orders of magnitude by using circular donor DNA containing flanking CRISPR target sites. By this means, we achieved an increase in rescue from ∼1% to up to 46% of the injected larvae. We found germ line transmission of the repaired allele in ∼10% (3/28) of fish raised to adulthood.

## RESULTS AND DISCUSSION

**Fig. 1.**
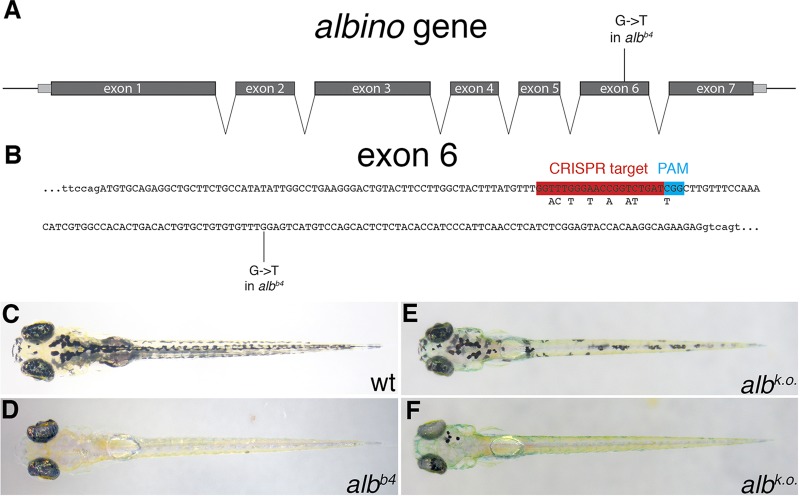
**CRISPR-mediated *alb* knockout.** (A) Schematic representation of the *alb* locus. The gene consists of seven exons (introns are not drawn to scale). The mutation *alb^b4^* introduces a premature stop codon into exon 6. (B) The sequence of exon 6 (in capitals). The CRISPR target site, PAM motif and the *alb^b4^* mutation are indicated; the SNPs introduced into the donor DNA fragments are shown beneath the CRISPR target site. (C-F) Dorsal views of larvae at 5 dpf: uninjected control wild type (C) and *alb^b4^* (D) larvae, and injected wild-type larvae with moderate (E) and good (F) *alb* knockout efficiency.

To test the feasibility of genome editing and the creation of knock-in alleles in zebrafish, we chose the *alb* phenotype as an easy and quick visible read-out that is independent of PCR methods, which, in our hands, are error prone and often produce false positive and false negative results. *alb* encodes a solute carrier (Slc45a2, Fig. 1A) that is responsible for the pH homoeostasis of melanosomes required for melanin production (Dooley et al., 2013b). Loss-of-function alleles cell-autonomously lead to unpigmented melanophores in the body and in the retinal pigment epithelium, a phenotype that is readily visible in early larvae from 2 days post fertilization (dpf) onwards, as well as in adult zebrafish (Fig. 1C,D) (Kelsh et al., 1996; Streisinger et al., 1986). A strong allele, *alb^b4^*, carries a nonsense mutation in exon 6 that leads to a truncated non-functional protein (Fig. 1A,B) (Dooley et al., 2013b). We first designed a single guide RNA (sgRNA) targeting exon 6 of the *alb* gene (Fig. 1B) and tested its efficiency in generating *alb* mutants when injected into wild-type one-cell stage embryos together with *Cas9* mRNA. We found that more than 95% of the injected larvae showed some defects in melanophore pigmentation, frequently with most or all melanophores being unpigmented (Fig. 1E,F). Most of these F_0_ individuals, when raised to adulthood, transmitted the *alb* knockout mutations to their offspring, demonstrating a very high efficiency of targeted mutagenesis in both soma and germ line. Similar efficiencies have been reported for the CRISPR/Cas9-mediated knockout of other genes in zebrafish (Jao et al., 2013).

**Fig. 2.**
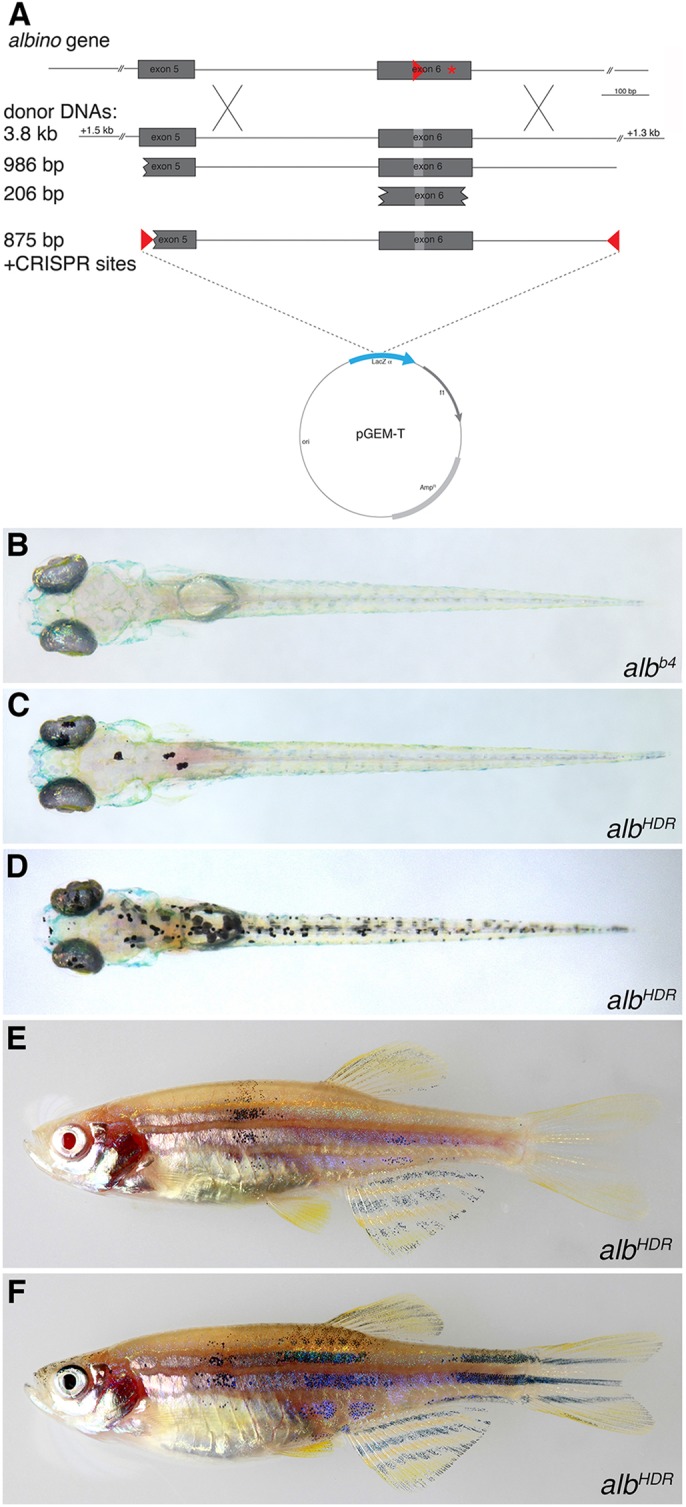
**Homology-directed repair at the *alb* locus.** (A) Donor DNAs used. The PCR fragments were also cloned into pGEM-T and injected as circular DNA molecules; the construct with the two CRISPR target sites is depicted. (B-D) Dorsal views of larvae 5 dpf: uninjected *alb^b4^* control (B), low efficiency repair using linear donor DNA (C) and high efficiency repair using circular donor DNA (D). (E,F) Two examples of adult F_0_ fish showing pigmented melanophores as a consequence of HDR in melanophore stem cells.

Next we co-injected linear donor DNA together with *Cas9* mRNA and sgRNA into *alb^b4^* embryos to test whether HDR can lead to repair of the mutation. As donor DNAs we used PCR products and gel-purified restriction fragments of different sizes, all containing exon 6 and in which we had destroyed the CRISPR target site by the introduction of silent mutations (Fig. 1B, Fig. 2). We found no rescue of the larval *alb^b4^* phenotype with a donor DNA fragment of only 206 bp. However, longer fragments of between 986 bp and 3.8 kb consistently gave rise to a few larvae with individual pigmented melanophores (∼1% of the larvae with 1-25 pigmented cells) (Fig. 2C, Table 1). The efficiency of repair is similar to the knock-in efficiencies reported previously (Hruscha et al., 2013), where 1.7% and 3.5% of all next generation sequencing reads showed correct integration of an HA tag into two different loci. We do not find differences in repair efficiencies between donor DNAs in which the 5′ end lies in intronic (as in the 3.8 kb donor DNA, Fig. 2A) or exonic (as in the 986 bp donor DNA, Fig. 2A) sequences; this indicates that the repair is most likely precise because short indels that might be tolerated in an intron would lead to frameshifts in the exon.

**Table 1.**
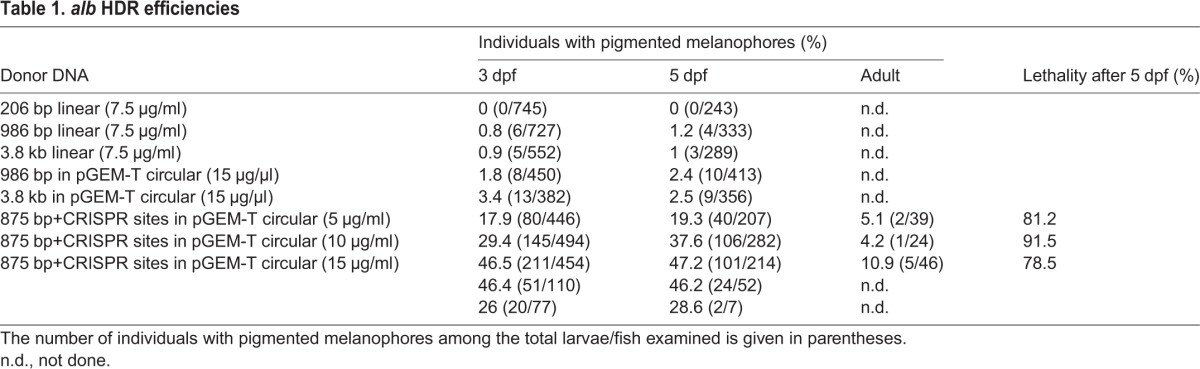
*alb* HDR efficiencies

The relatively low efficiency of HDR that we observed could be due to the very low concentrations of donor DNA that had to be used (7.5 ng/µl) owing to the inherent toxicity of linear DNA. We therefore injected circular donor DNAs (PCR products cloned into the bacterial vector pGEM-T) at higher DNA concentrations of 15 ng/µl and obtained slightly better efficiencies, with ∼2.5% of the larvae showing some cells with melanin production indicating a repaired *alb* locus (Table 1). To further increase the efficiency we constructed the donor DNA as a circular plasmid in which an 853 bp DNA fragment derived from the *alb* gene is flanked by two CRISPR target sites. This fragment can be linearized in the cells to provide the linear template for HDR once the Cas9 protein is translated from the co-injected mRNA (Fig. 2A). This strategy led to a considerable increase in the efficiency of rescue, resulting in up to 46% of larvae with some pigmented melanophores at 3 dpf. Most larvae still had only relatively few pigmented melanophores (1-25 cells), but in several instances we found up to 150 pigmented cells per larva (Fig. 2D). At this efficiency of (somatic) rescue, the lethality is rather high, with only ∼10% of 3-day-old larvae reaching adulthood (Table 1). However, of the adult fish we obtained, 10% displayed some melanophore pigmentation, demonstrating that some of the small set of melanophore stem cells (Dooley et al., 2013a) were affected in these cases (Fig. 2E,F, Table 1). Of 28 adult fish that gave rise to F_1_ progeny, three (∼10%) produced pigmented larvae of wild-type appearance with frequencies between 3% and 24% (Table 2). Sequence analysis confirmed that these larvae carried the repaired version of the *alb* gene including the SNPs that we introduced to change the CRISPR target site in exon 6 of the gene (supplementary material Fig. S1).

**Table 2.**
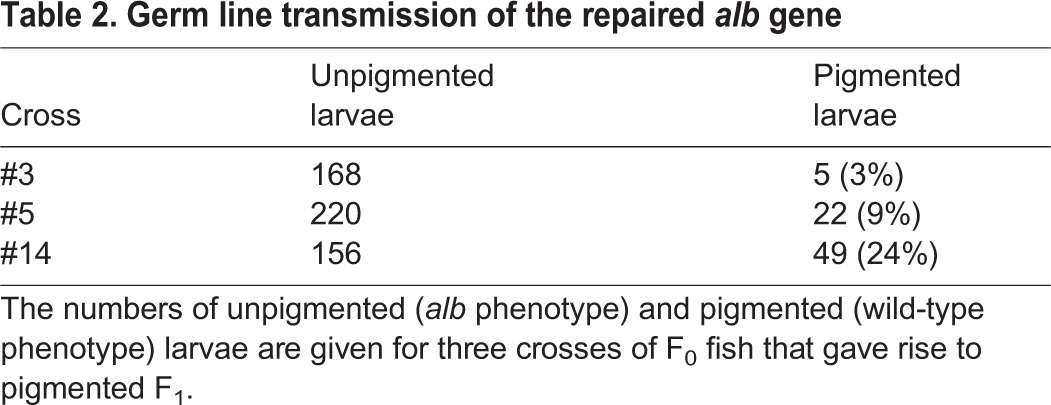
**Germ line transmission of the repaired** *alb* gene

Taken together, we have shown that precise genome editing in zebrafish is possible with very high efficiency using circular donor DNA of less than 1 kb in length flanked by CRISPR target sites. This analysis provides an important proof of principle for genomic modifications in this important model for vertebrate biomedical research; we expect that precise genome editing in zebrafish is possible at other loci with similar efficiencies.

## MATERIALS AND METHODS

### Zebrafish

We used wild type (TÜ) and *alb[b4]* (Dooley et al., 2013b) mutant zebrafish (*Danio rerio*). All experiments with zebrafish were performed in accordance with the guidelines of the Max-Planck-Society and approved by the Regierungspräsidium Tübingen, Baden-Württemberg, Germany (Aktenzeichen: 35/9185.46).

### Preparation of *Cas9* RNA and sgRNAs

PCR amplifications were performed with KOD Hot Start DNA polymerase (Novagen).

The *Cas9* coding sequence was PCR amplified from plasmid pMLM3613 (Addgene) using primers 1106 and 1107 (Table 3). Single A-overhangs were added using Taq polymerase and the fragment was cloned into pGEM-T Easy (Promega) and sequenced. The insert was then excised with *Bgl*II and *Xba*I and cloned into pCS2 via *Bam*HI and *Xba*I. This plasmid was linearized with *Not*I, and *in vitro* transcription and polyadenylation were carried out with the mMessage mMachine SP6 Kit and polyA Tailing Kit (both from Ambion) according to the manufacturer's instructions. The RNA was purified by precipitation with isopropanol.

**Table 3.**
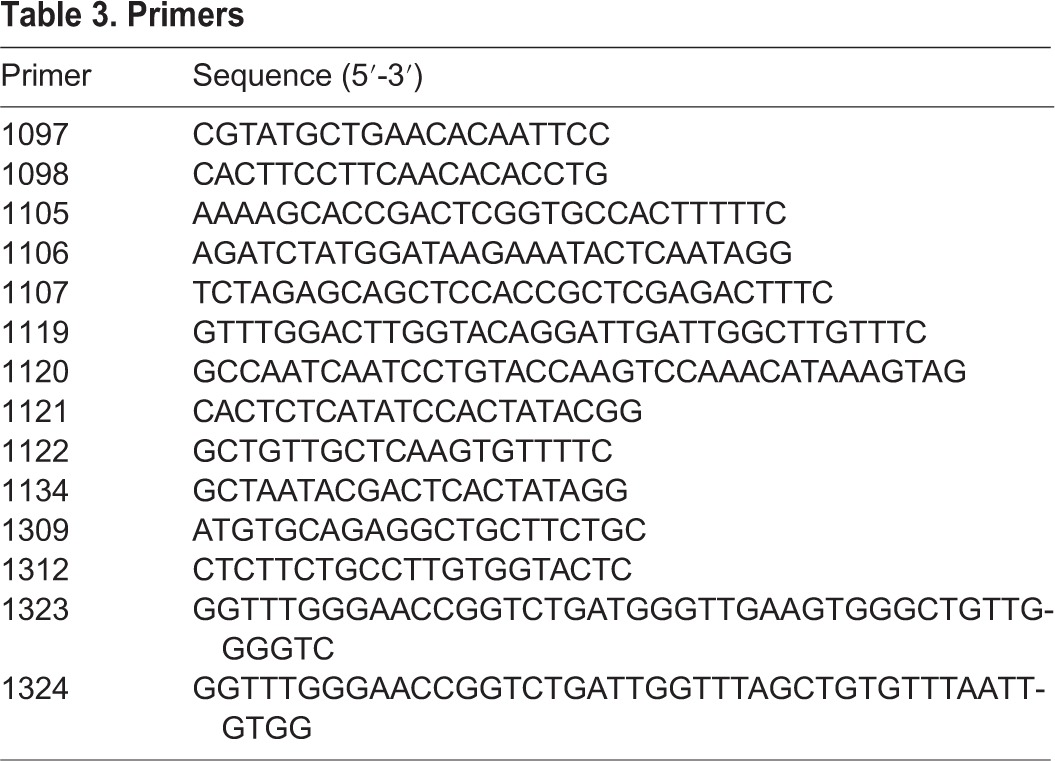
**Primers**

CRISPR target sites were identified using the ZiFiT Targeter (http://zifit.partners.org/ZiFiT/); the selected oligonucleotides were cloned into pDR274 (Addgene) linearized with *Bsa*I. The templates for *in vitro* transcription of sgRNAs were produced by PCR using primers 1105 and 1134 (Table 3). The *in vitro* transcription was carried out using 150-200 ng template and the MegaScript T7 Kit (Ambion). The RNA was purified by precipitation with isopropanol.

### Preparation of HDR templates and donor DNAs

DNA templates for HDR were generated by PCR from genomic DNA of wild-type (TÜ or WIK) fish. In a first step, primers 1121 and 1122 were used. A-overhangs were added and the PCR product was cloned into pGEM-T Easy. The CRISPR target site was modified by single-step mutagenesis using primers 1119 and 1120. Linear templates for HDR were generated from the resulting plasmid by PCR with primers 1097, 1098, 1121, 1122, 1309 and 1312. Primer sequences are listed in Table 3.

Donor DNA flanked by CRISPR target sites was generated by PCR with primers 1323 and 1324 (Table 3). A-overhangs were added and the PCR product was cloned into pGEM-T Easy.

### Injection and genotyping

One-cell stage zebrafish embryos were injected with ∼2-3 nl of a solution containing 250 ng/µl *Cas9* mRNA, 15 ng/µl sgRNA and 5-50 ng/µl template DNA.

For PCR amplification of the repaired *alb* locus from F_1_ larvae, the tissue samples were incubated in TE supplemented with 5% Chelex-100 (BioRad) and 10 µg/ml Proteinase K (Roche) for 4 h at 55°C and 10 min at 95°C and then stored at 4°C. 1 µl of the supernatant was used as template in a standard 25 µl PCR with primers 1097 and 1098. The PCR product was cloned into pGEM-T Easy and sequenced with M13 forward and M13 reverse primers (Invitrogen).

## Supplementary Material

Supplementary Material
