# Supplementary material for: Precise and efficient genome editing in zebrafish using the CRISPR/Cas9 system
Source: Development. 2014 Dec 15;141(24):4827–30. doi: 10.1242/dev.115584 (PMC4299274; doi:10.1242/dev.115584)
Supplement: Supplementary Material [file supp_141_24_4827__index.html]

Supplementary Material 

# Precise and efficient genome editing in zebrafish using the CRISPR/Cas9 system

## DEV115584 Supplementary Material

**Files in this Data Supplement:**

- Supplementary Material
