# Supplementary material for: Precise and efficient genome editing in zebrafish using the CRISPR/Cas9 system
Source: Development. 2014 Dec 15;141(24):4827–30. doi: 10.1242/dev.115584 (PMC4299274; doi:10.1242/dev.115584)
Supplement: Supplementary Material [file supp_dev.115584_DEV115584supp.pdf]

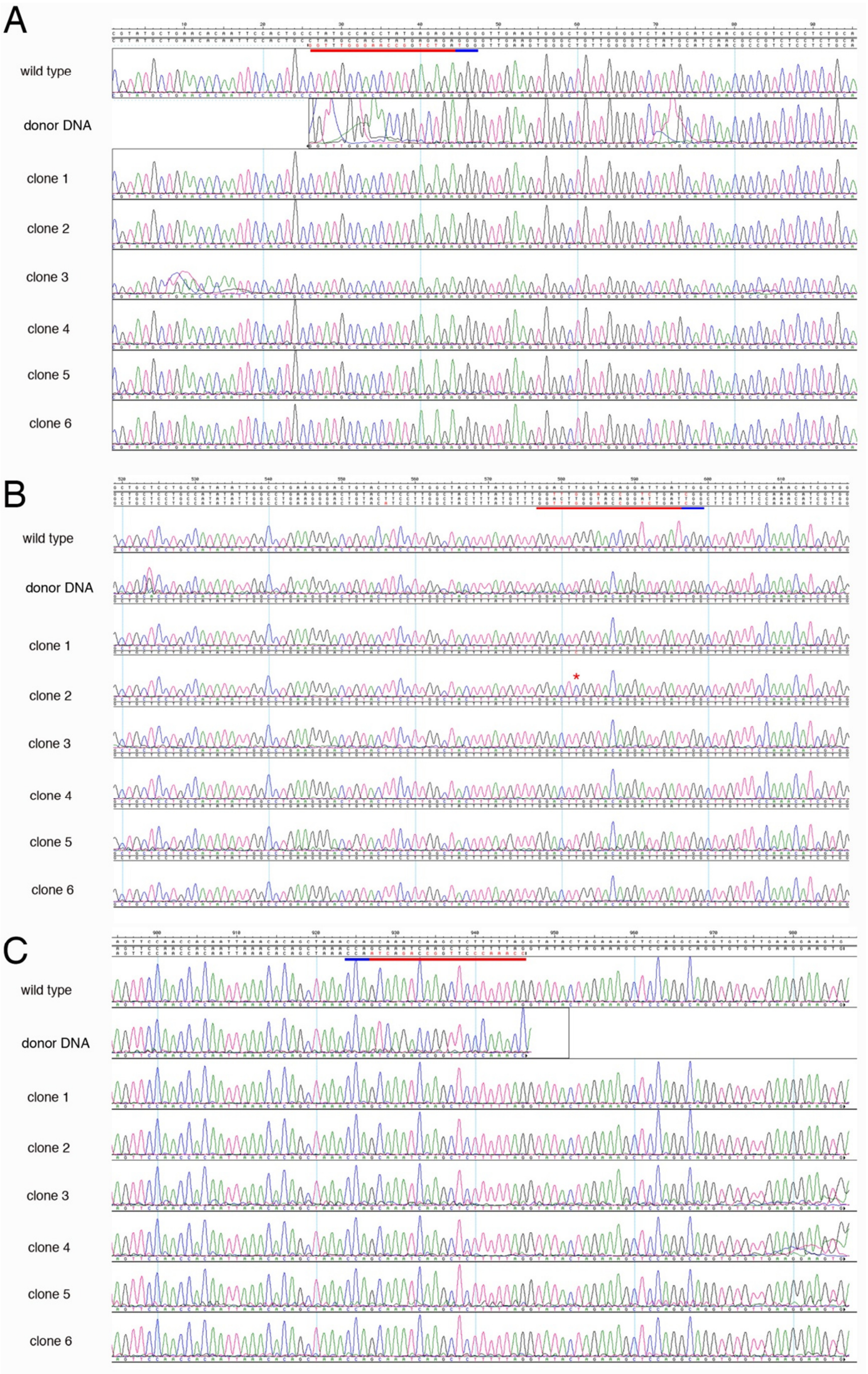
A
wild type
donor DNA
clone 1
clone 2
clone 3
clone 4
clone 5
clone 6
B
wild type
donor DNA
clone 1
clone 2
clone 3
clone 4
clone 5
clone 6
C
wild type
donor DNA
clone 1
clone 2
clone 3
clone 4
clone 5
clone 6

Supplementary Figure 1. Sequence analysis of the repaired *alb* locus.

The complete region of the donor DNA was sequenced from two independent clones for each of the three lines with pigmented F1 larvae. The sequences were compared to wild type and to the donor DNA construct. The 5' end is shown in (A) all clones are identical to the wild type sequence. In (B) the region of exon 6 with the CRISPR target site is shown. All six clones show the changes that were introduced into the donor DNA construct. Clone 2 has an additional change at position 582 (asterisk), this could be a PCR error or a change that occurred in the fish, as it is a synonymous change. The 3' end is shown in (C), again the sequences of all six clones are identical to wild type and different from the donor DNA construct. The CRISPR target sites are underlined in red, the PAM motif in blue.
